# Association of JMJD2B and Hypoxia-Inducible Factor 1 Expressions with Poor Prognosis in Osteosarcoma

**DOI:** 10.1155/2020/2563208

**Published:** 2020-07-31

**Authors:** Xujian Liu, Qianqian Zhang, Yi Zhao, Jianjun Xun, Hongzeng Wu, Helin Feng

**Affiliations:** ^1^Department of Orthopedics, The Fourth Hospital of Hebei Medical University, 12 Health Road, Shijiazhuang, Hebei 050011, China; ^2^Department of Gynecology, Hebei Medical University Second Affiliated Hospital, 215 Heping Road, Shijiazhuang, Hebei 050011, China

## Abstract

**Background:**

JMJD2B has been reported to be implicated in malignant tumors. This study is aimed at exploring the expression and prognostic significance of JMJD2B in osteosarcoma and its association with hypoxia-inducible factor 1 (HIF1).

**Methods:**

The histopathological and clinical characteristics were retrospectively reviewed from 53 osteosarcoma patients. JMJD2B and HIF1 were examined by immunohistochemical staining of paraffin-embedded osteosarcoma samples, and their association with clinical characteristics was examined by Spearman's test. Overall survival was examined by Kaplan-Meier analysis, and prognostic factors were identified by univariate and multivariate regression analyses.

**Results:**

JMJD2B and HIF1 expression levels were both significantly associated with Enneking stage, distant metastasis, and neoadjuvant chemotherapy, and the JMJD2B and HIF1 expressions were positively correlated (*p* < 0.001, *R* = 0.752). In addition, univariate analysis showed that the expression of both JMJD2B and HIF1 was significantly associated with overall survival, but multivariate analysis showed that only JMJD2B expression was significantly associated with overall survival in osteosarcoma patients.

**Conclusions:**

JMJD2B and HIF1 expression levels show significant correlation with osteosarcoma progression, and JMJD2B could predict poor prognosis of osteosarcoma patients.

## 1. Introduction

Osteosarcoma (OS) is a common malignant bone tumor [1]. Despite curative resection of primary tumor, around 40% of OS patients develop isolated pulmonary metastases during the clinical course of OS [1]. There are limited novel therapeutic options, and the survival rate of patients with OS has not improved in the past decades. Better prognostic factors are important to improve the survival of patients with refractory OS.

Hypoxia-inducible factor 1 (HIF1) plays an important role in the response to low oxygen of cancer cells [2–4]. HIF1 help cancer cells adapt to hypoxia and promotes tumorigenesis [5]. HIF1 is overexpressed in many solid tumors, including OS, leading to unfavorable clinical outcome and poor survival [5]. Jumonji domain-containing protein 2B (JMJD2B) is a member of the JMJD2 family of histone demethylases and is directly regulated by HIF1 [6]. JMJD2B contains the catalytic JmjC domain to demethylate tri- and di-methylated lysine 9 (H3K9me3/2) on histone H3 [7]. JMJD2B is overexpressed in a variety of cancers including the liver, colon, lung, and gastric cancer as well as acute myeloid leukemia [8–11]. However, the association of the HIF1 and JMJD2B expressions and their significance in OS patients remain unclear. In this study, we aimed to explore the expression and prognostic significance of JMJD2B and HIF1 in OS.

## 2. Materials and Methods

### 2.1. Subjects

This study was approved by the ethics committee of the Fourth Hospital of Hebei Medical University, and all subjects signed a written informed consent. OS samples were collected from 53 patients who visited the Fourth Hospital of Hebei Medical University between 2005 and 2013. Their histopathological and clinical characteristics were collected from medical records. No patients received radiotherapy or chemotherapy before surgery. The patients were grouped based on the age, gender, tumor size (<5 cm and ≥5 cm), histological grade, Enneking stage (I-III), and neoadjuvant chemotherapy status.

### 2.2. Immunohistochemistry Analysis

Immunohistochemical staining was performed on OS tissues using HIF1 antibodies (1 : 100; Abcam, USA) and JMJD2B antibodies (1 : 100; Abcam, USA) following the protocols described previously [12]. The number of positively stained cells in five randomly selected fields was analyzed by three pathologists blindly. The staining intensity was evaluated as follows: 0, no staining; 1, beige; 2, darker beige; and 3, tan. The staining extent was scored as follows: 0, 0% stained; 1, 1% to 25% stained; 2, 26% to 50% stained; and 3, 51% to 100% stained. The final scores were calculated by multiplying intensity score and extent score and judged as follows: 0 to 2, low staining (-/+); 3 to 5, medium staining (++); and 6 to 9, high staining (+++).

### 2.3. Statistical Analysis

Potential prognostic factors were analyzed by *χ*^2^ test. Correlations of histological and clinical variables were analyzed by Spearman's rho test. Univariate and multivariate analyses were conducted using Cox proportional hazards regression analysis. Survival curves were plotted by the Kaplan-Meier method. All statistical analyses were conducted using SPSS 25.0 software, and significance was set at *p* < 0.05.

## 3. Results

### 3.1. Characteristics of OS Patients

The characteristics of OS patient were summarized in Table 1, their mean age was 35 years (range, 9-75 years), and median overall survival was 21 months (range, 2-91 months).

### 3.2. Correlation of HIF1 and JMJD2B Expressions with Clinicopathological Characteristics of OS Patients

**Figure 1:**
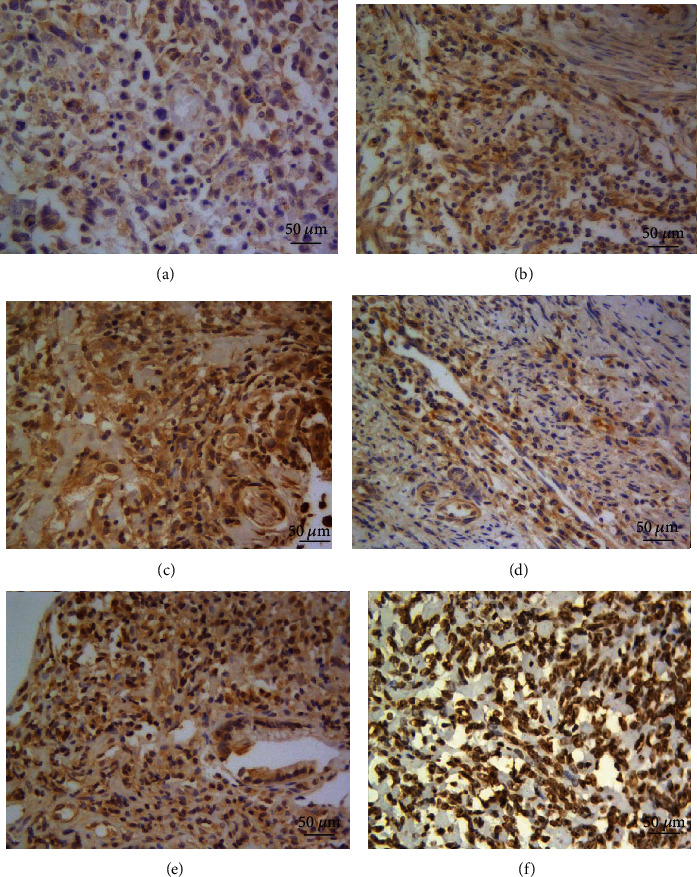
Representative immunohistochemical staining of HIF1 and JMJD2B. (a) Low expression of HIF1 in OS. (b) Moderate expression of HIF1 in OS. (c) High expression of HIF1 in OS. (d) Low expression of JMJD2B in OS. (e) Moderate expression of JMJD2B in OS. (f) High expression of JMJD2B in OS. Cells with positive expression were stained brown.

Correlation of the HIF1 and JMJD2B expressions with clinicopathological characteristics of OS patients was presented in Table 1. Representative images of HIF1 and JMJD2B staining were shown in Figure 1. Both HIF1 and JMJD2B staining were primarily in the nucleus, but the staining scores varied from low to high among OS tissues. Specifically, the HIF1 expression was low in 17.0% (9/53), moderate in 28.3% (15/53), and high in 54.7% (29/53) of OS tissues, while the JMJD2B expression was low in 13.2% (7/53), moderate in 34.0% (18/53), and high in 52.8% (28/53) of OS tissues. Both HIF1 and JMJD2B showed significant association with distant metastasis (*p* < 0.001), Enneking stage (*p* < 0.001), and treatment with neoadjuvant chemotherapy (*p* < 0.001) but showed no significant association with other clinicopathological parameters. Notably, we observed a significant positive correlation between the HIF1 and JMJD2B expressions (*p* < 0.001) (Table 2).

### 3.3. Correlation of HIF1 and JMJD2B Expressions with Poor Overall Survival of OS Patients

**Figure 2:**
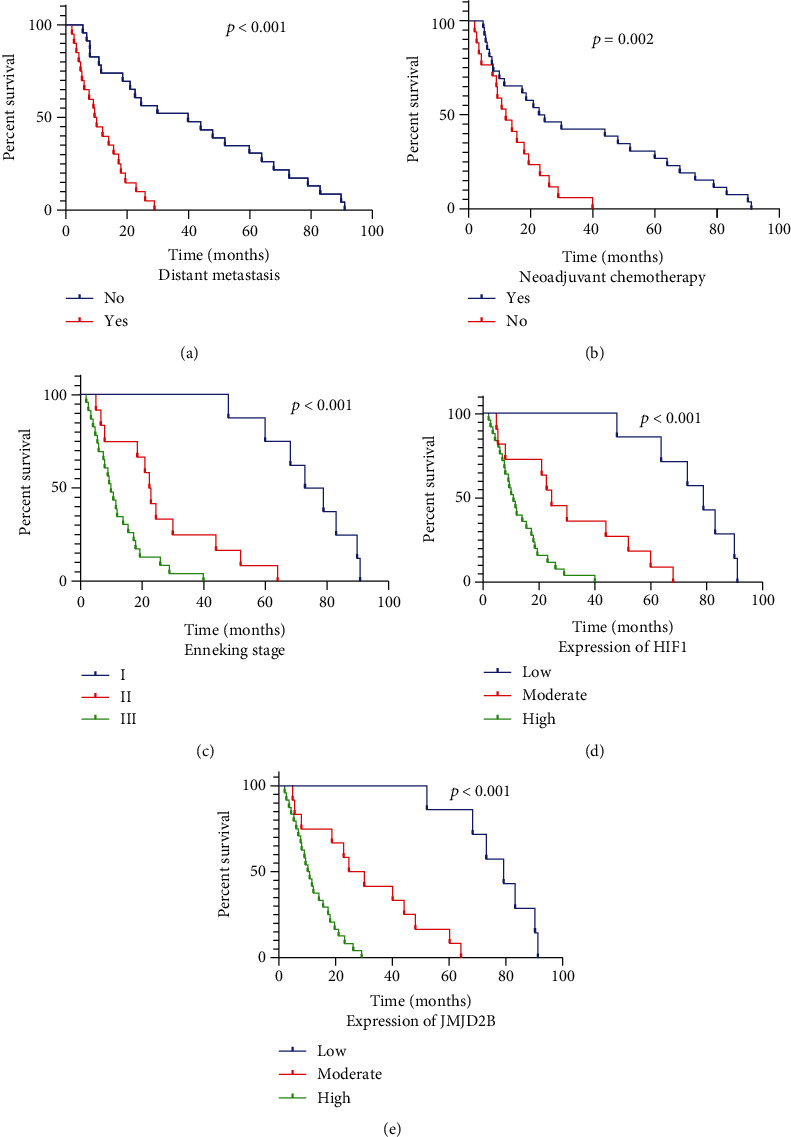
Overall survival curves for OS patients. Association of overall survival with (a) distant metastasis, (b) neoadjuvant chemotherapy, (c) Enneking stage, (d) HIF1 expression, and (e) JMJD2B expression. *p* values were determined by comparing survival distributions using the log-rank test.

Table 3 showed the results of univariate regression analysis of overall survival. The age, gender, tumor size, and histological grade were not significant predictors of overall survival, but distant metastasis (*p* < 0.001), treatment with neoadjuvant chemotherapy (*p* < 0.001), and higher Enneking stage (*p* < 0.001) predicted shorter overall survival (Figure 2). Furthermore, higher HIF1 (*p* < 0.001) and JMJD2B (*p* = 0.001) expressions showed significant association with shorter overall survival (Table 3, Figure 2).

### 3.4. Higher JMJD2B Expression Predicted Poor Survival of OS Patients

Table 4 showed the results of multivariate regression analysis of factors associated with overall survival revealed by univariate analysis. For multivariate analysis, only the JMJD2B expression (*p* < 0.001), neoadjuvant chemotherapy (*p* < 0.001), and Enneking stage (*p* < 0.001) were identified as significant prognostic factors in OS patients. OS patients with high Enneking stage, history of neoadjuvant chemotherapy, and high JMJD2B expression had significantly increased risk of mortality (log-rank *p* < 0.001; Figure 2).

## 4. Discussion

To our knowledge, this is the first study to explore the expression pattern and prognostic significance of HIF1 and JMJD2B in OS patients. We found that higher HIF1 and JMJD2B levels showed a significant correlation with unfavorable clinical variables of OS patients. In addition, the HIF1 and JMJD2B expressions were positively correlated, and both HIF1 and JMJD2B were positively correlated with Enneking stage and distant metastasis. Moreover, univariate analysis showed a significant association of high HIF1 and JMJD2B expression levels with shorter overall survival in OS patients, and the JMJD2B expression was an independent prognostic indicator of OS patients. Overall, these results suggest that JMJD2B is a significant prognostic factor of OS.

HIF1 is overexpressed in a variety of solid tumors [13]. Moreover, our previous study indicated that HIF1 was overexpressed and possibly mediated hypoxia-induced autophagic activation in human OS tissues [14]. In this study, we confirmed that HIF1 was highly expressed in OS tissues and associated with poor survival, in agreement with previous findings that high HIF1 expression indicated poor outcomes in several types of cancer [15, 16].

JMJD2B demethylates methylation marks on H3K9 and H3K36, resulting in the activation of transcription [17]. HIF1 can regulate the transcription of JMJD2B due to the presence of hypoxia response elements in its promoter region [6]. JMJD2B also regulates cell proliferation and promotes bladder and lung cancer cell growth by modulating cyclin-dependent kinase 6 [18–20]. In addition, a previous study showed that significant upregulation of JMJD2B in tumor tissues promoted the expression of fibroblast growth factor 2 and became a risk factor for the development of OS [11]. In this study, we confirmed that JMJD2B could be a new prognostic marker of OS. However, the mechanisms underlying the regulation of HIF1 and JMJD2B in OS remain to be elucidated.

The present study has several limitations. First, immunohistochemical analysis is semiquantitative and needs further validation by real-time PCR. Second, our sample size was small, and our conclusion should be validated in larger samples.

In conclusion, HIF1 and JMJD2B are highly expressed in OS tissues and associated with unfavorable clinical characteristics and poor prognosis of OS patients. JMJD2B could a new clinical prognostic factor for OS patients.

## Figures and Tables

**Table 1:** Clinicopathological variables and HIF1 and JMJD2B expressions in OS patients.

Characteristics				HIF1			JMJD2B			
			Low (%)	Moderate (%)	High (%)	*p*	Low (%)	Moderate (%)	High (%)	*p*
Gender	Female	23	4 (17.4)	5 (21.7)	14 (60.9)	0.555	2 (8.7)	8 (34.8)	13 (56.5)	0.471
	Male	30	5 (16.7)	10 (33.3)	15 (50.0)		5 (16.7)	10 (33.3)	15 (50.0)	

Age	<20 years	22	5 (22.7)	5 (22.7)	12 (54.5)	0.752	9 (9.1)	9 (40.9)	11 (50.0)	0.953
	≥20 years	31	4 (12.9)	10 (32.3)	17 (54.8)		5 (16.1)	9 (29.0)	17 (54.8)	

Tumor size	<5 cm	24	5 (20.8)	8(333)	11(458)	0. 253	4 (167)	10 (41.7)	10 (41.7)	0.155
	≥5 cm	29	4 (13.8)	7 (24.1)	18 (62.1)		3 (10.3)	8 (27.6)	18 (62.1)	

Histologic grade	Well differentiated	18	2 (11.1)	7 (38.9)	9 (50.0)	0. 965	3 (16.7)	7 (38.9)	8 (44.4)	0.757
	Moderately differentiated	19	3 (15.8)	5 (26.3)	11 (57.9)		3 (15.8)	5 (26.3)	11 (57.9)	
	Poorly differentiated	16	4 (25.0)	3 (18.8)	9 (56.3)		1 (6.3)	6 (27.5)	9 (56.3)	

Distant metastasis^∗^	No	30	9 (30.0)	13 (43.3)	8 (26.7)	<0.001	7 (23.3)	16 (53.3)	7 (23.3)	<0.001
	Yes	23	0 (0.0)	2 (8.7)	21 (91.3)		0 (0.0)	2 (8.7)	21 (91.3)	

Neoadjuvant chemotherapy^∗^	Yes	32	8 (25.0)	15 (46.9)	9 (28.1)	<0.001	7 (21.9)	16 (50.0)	9 (28.1)	<0.001
	No	21	1 (4.8)	0 (0.0)	20 (95.2)		0 (0.0)	2 (9.5)	19 (90.5)	

Enneking stage^∗^	I	10	8 (80.0)	2 (20.0)	0 (0.0)	<0.001	6 (60.0)	3 (30.0)	1 (10.0)	<0.001
	II	18	1 (5.6)	11 (61.1)	6 (33.3)		1 (5.6)	10 (55.6)	7 (38.9)	
	III	25	0 (0.0)	2 (8.0)	23 (54.7)		0 (0.0)	5 (20.0)	20 (80.0)	

The Spearman-rho test was used. ^∗^*p* < 0.05.

**Table 2:** Correlation of HIF1 and JMJD2B expressions.

Characteristics			JMJD2B		*p*
		Low (%)	Moderate (%)	High (%)	
	Low	5 (55.6)	3 (33.3)	1 (11.1)	<0.001
HIFI	Moderate	2 (13.3)	11 (73.3)	2 (13.3)	
	High	0 (0.0)	4 (13.8)	25 (86.2)	

The Spearman-rho test was used. ^∗^*p* < 0.05.

**Table 3:** Univariate Cox proportional regression analysis for the association of clinicopathological factors with overall survival of OS patients.

Characteristics			HR	OS	*p*
				95% CI	
Gender	Female	23	1		0.214
	Male	30	0.677	0.366-1.252	

Age	<20 years	22	1		0.865
	≥20 years	31	0.948	0.511-1.759	

Tumor size	<5 cm	24	1		0.492
	≥5 cm	29	1.240	0.671-2.291	

Histologic grade	Well differentiated	18	1		0.988
	Moderately differentiated	19	0.998	0.478-2.080	
	Poorly differentiated	16	1.053	0.499-1.053	

Distant metastasis^∗^	No	30	1		<0.001
	Yes	23	5.311	2.403-11.736	

Neoadjuvant chemotherapy^∗^	Yes	32	1		0.002
	No	21	3.118	1.522-6.389	

Enneking stage^∗^	I	10	1		<0.001
	II	9	12.605	2.643-60.109	
	III	25	51.909	9.949-270.831	

HIF1	Low	9	1		<0.001
	Moderate	15	10.589	2.230-50.275	
	High	29	44.344	8.280-237.493	

JMJD2B	Low	7	1		<0.001
	Moderate	18	20.018	2.470-162.224	
	High	28	110.895	11.857-1037.165	

OS: overall survival; HR: hazard ratio; 95% CI: 95% confidence interval. ^∗^*p* < 0.05.

**Table 4:** Multivariate Cox proportional regression analysis for the association of clinicopathological factors with overall survival of OS patients.

Characteristics	OS		
	HR	95% CI	*p*
Neoadjuvant chemotherapy^∗^	0.424	0.185-0.972	0.043
Enneking stage^∗^	4.069	1.685-9.826	0.002
JMJD2B^∗^	4.002	1.492-10.735	0.006

OS: overall survival; HR: hazard ratio; 95% CI: 95% confidence interval. ^∗^*p* < 0.05.

## Data Availability

All data and material are available upon request.
